# Human milk oligosaccharides differentially support gut barrier integrity and enhance Th1 and Th17 cell effector responses *in vitro*

**DOI:** 10.3389/fimmu.2024.1359499

**Published:** 2024-03-06

**Authors:** Erik Juncker Boll, Daniel Villalba Lopez, Mandy Terne, Sara Hessing, Katja Parschat, Stina Rikke Jensen

**Affiliations:** ^1^ Chr. Hansen A/S, Applied HMOs, Hoersholm, Denmark; ^2^ Chr. Hansen HMO GmbH, Rheinbreitbach, Germany

**Keywords:** human milk oligosaccharides, intestinal barrier integrity, dendritic cells, macrophages, T cells, cytokines

## Abstract

Human milk oligosaccharides (HMOs) can modulate the intestinal barrier and regulate immune cells to favor the maturation of the infant intestinal tract and immune system, but the precise functions of individual HMOs are unclear. To determine the structure-dependent effects of individual HMOs (representing different structural classes) on the intestinal epithelium as well as innate and adaptive immune cells, we assessed fucosylated (2′FL and 3FL), sialylated (3′SL and 6′SL) and neutral non-fucosylated (LNT and LNT2) HMOs for their ability to support intestinal barrier integrity, to stimulate the secretion of chemokines from intestinal epithelial cells, and to modulate cytokine release from LPS-activated dendritic cells (DCs), M1 macrophages (MØs), and co-cultures with naïve CD4^+^ T cells. The fucosylated and neutral non-fucosylated HMOs increased barrier integrity and protected the barrier following an inflammatory insult but exerted minimal immunomodulatory activity. The sialylated HMOs enhanced the secretion of CXCL10, CCL20 and CXCL8 from intestinal epithelial cells, promoted the secretion of several cytokines (including IL-10, IL-12p70 and IL-23) from LPS-activated DCs and M1 MØs, and increased the secretion of IFN-γ and IL-17A from CD4^+^ T cells primed by LPS-activated DCs and MØs while reducing the secretion of IL-13. Thus, 3′SL and 6′SL supported Th1 and Th17 responses while reducing Th2 responses. Collectively, our data show that HMOs exert structure-dependent effects on the intestinal epithelium and possess immunomodulatory properties that confer benefits to infants and possibly also later in life.

## 1 Introduction

Human milk oligosaccharides (HMOs) are the third most abundant bioactive component in human breastmilk. More than 150 structurally distinct HMOs have been identified, which are generally divided into three structural groups: neutral fucosylated, neutral non-fucosylated, and acidic (sialylated) HMOs (1). HMOs benefit infant health by modulating the gut microbiome (2–4), strengthening intestinal barrier integrity and function (5, 6), immunomodulation (7, 8), and supporting cognitive development (9, 10). Most HMOs are undigested when they reach the lower gut, where they are fermented by the intestinal microbiome (11). However, a small fraction of HMOs may be absorbed into the systemic circulation (12, 13), indicating that the biological functions of HMOs extend beyond the gut and microbes.

The intestinal epithelium is necessary for gut homeostasis because the cells and overlying mucus layer form a barrier separating the luminal contents from the submucosa and systemic circulation (14, 15). When the intestinal barrier is impaired, luminal components, including bacteria, may translocate into the submucosal tissues causing mucosal and systemic inflammation, and may contribute to gastrointestinal and/or systemic diseases (16). An impaired intestinal barrier is a particular concern in preterm infants because it increases the risk of life-threatening necrotizing enterocolitis (17). Term infants are born with an immature and more permeable intestine, but intestinal integrity normally increases during the first weeks of life (18). Intestinal permeability decreases more rapidly in breastfed compared to formula-fed infants (18, 19), suggesting that breastmilk contains components that contribute to the maturation of the intestine. HMOs have been shown to support the maturation of the intestine *in vitro* and *in vivo* by increasing barrier integrity and stimulating mucin expression (5, 20). However, the effect of individual HMO structures on the integrity of the intestinal epithelial barrier is unclear.

Intestinal cells closely interact with the local submucosal immune compartment e.g., by releasing chemokines and cytokines that recruit and/or activate immune cells, including dendritic cells (DCs), macrophages (MØs) and T cells (14). DCs are specialized antigen-presenting cells (APCs) that regulate innate and subsequent adaptive immune responses. Immature DCs in the lamina propria sample the luminal contents, and if they sense pathogen-associated molecular patterns such as lipopolysaccharides (LPS), they become activated, undergo maturation, adjust their cytokine environment, and migrate to lymph nodes where they encounter naïve T cells and induce specific effector T cell responses (21). Similarly, MØs can present antigens to T cells and initiate adaptive immune responses (22, 23). A subtype of MØs (classical or M1 MØs) possess microbicidal activity and promote strong Th1 responses (23, 24). Activated DCs and MØs engage with naïve CD4^+^ T cells and create a cytokine milieu that drives the differentiation of the latter into specific effector T helper (Th) cells, including Th1, Th2 and Th17 subtypes. In the presence of interleukin (IL)-12, naïve CD4^+^ T cells differentiate towards Th1 rather than the Th2 direction. Th1 cells produce large amounts of interferon (IFN)-γ, which promotes cell-mediated immunity. In the presence of IL-4, naïve CD4^+^ T cells favor differentiation into Th2 cells that produce large amounts of IL-4, IL-5, and IL-13, triggering antibody-mediated responses and protection against extracellular parasites (24, 25). Cytokines such as IL-1β, IL-6, IL-23, and tumor necrosis factor (TNF)-α support the differentiation of Th17 cells (26–28), which produce large amounts of IL-17A and drive host-mediated responses against extracellular bacteria, particularly at epithelial surfaces (29).

During pregnancy, the immune system at the maternal–fetal interface is skewed towards a Th2 phenotype, which ensures that the developing fetus is protected from the maternal immune response, hence preventing rejection (30). This Th2 skewing persists into early infanthood, but the excessive production of Th2 cytokines during this period may enhance the risk of allergies later in life (31). Therefore, improved Th1 immunity and the induction of tolerance in early life is important to ensure the proper development of adaptive immunity (31, 32). Pooled HMOs isolated from breastmilk have been shown to inhibit Th1 differentiation in a co-culture model of human blood monocyte-derived DCs and naïve CD4^+^ T cells (33). In another study, the acidic fraction of pooled breastmilk-derived HMOs increased IFN-γ production from cord blood-derived mononuclear cells (34). Using a co-culture model of intestinal epithelial cells and peripheral blood-derived mononuclear cells (PBMCs), 2′-fucosyllactose (2′FL) and 3-fucosyllactose (3FL) were found to inhibit Th2 differentiation (35). Furthermore, the acidic HMO 3′-sialyllactose (3′SL), but not the structurally similar 6′-sialyllactose (6′SL), increased the intestinal expression of *IL-17A* mRNA in a mouse model of colitis (36).

Although the studies discussed above demonstrated direct effects of HMOs on the intestinal barrier and immune cell responses, the specific role of individual HMOs in these contexts remains poorly understood. Here, we investigated the structure-dependent *in vitro* effects of fucosylated (2′FL and 3FL), sialylated (3′SL and 6′SL) and neutral non-fucosylated (lacto-*N*-tetraose (LNT) and lacto-*N*-triose II (LNT2)) HMOs on intestinal epithelial cells, monocultures of DCs and M1 MØs, and CD4^+^ T cells primed with DCs or MØs cultured with HMOs. Our data suggest that primarily fucosylated and neutral non-fucosylated HMOs trigger a dose-dependent increase in intestinal barrier integrity, whereas sialylated HMOs exert immunomodulatory effects by influencing T cell effector functions and by enhancing the secretion of chemokines and cytokines from the epithelium and immune cells, respectively.

## 2 Materials and methods

### 2.1 Human milk oligosaccharides

The HMOs used in this study (2′FL, 3FL, 3′SL, 6′SL, LNT and LNT2) were produced by Chr. Hansen HMO GmbH, Rheinbreitbach, Germany. HMO stock solutions were dissolved in water or in cell culture media (see Section 2.2). Endotoxin levels are presented in **Table 1**.

**Table 1:** Endotoxin levels in the tested HMOs according to Ph. Eur. Method 2.6.14: Test for Bacterial Endotoxins (LAL test).

HMO	Endotoxin (EU/mg)
2′FL	0.009
3FL	< 0.005
3′SL	< 0.005
6′SL	< 0.005
LNT	< 0.005
LNT2	Not known

EU = unit of endotoxin activity.

### 2.2 Cell culture

The human epithelial intestinal cancer cell line Caco-2 (ACC 169, DSMZ, passages 5–20) was maintained in MEM, GlutaMAX Supplement (Gibco, Cat# 41090036) supplemented with 1% non-essential amino acids (Merck Life Science; Cat# M7145), 1% penicillin-streptomycin (10,000 U/mL) (Gibco; Cat# 15140122), and 10% heat-inactivated fetal bovine serum (FBS; Gibco; Cat# 10500064) at 37°C in a 5% CO_2_ atmosphere. The cells were seeded on transwells (Corning; Cat# CLS3460) at a density of 1×10^5^ cells/insert. The culture medium was changed every 3–4 days for 21–23 days, by which time the cells had reached a confluent and differentiated state. HT29 cells (ACC 299, DSMZ, passages 5–20) were cultured in McCoy’s 5A (modified) medium GlutaMAX Supplement (Gibco; Cat# 36600021) with 1% penicillin-streptomycin and 10% heat-inactivated FBS. For analysis, cells were seeded at a density of 5×10^4^ cells/well in 48-well culture plates (TPP; Cat# 92048).

### 2.3 Transepithelial electrical resistance assays

After 21–23 days, transwells with Caco-2 cells were transferred into a CellZscope2 device (NanoAnalytics). When a stable transepithelial electrical resistance (TEER) baseline was reached (≥ 300 Ω·cm^2^, > 12 h), HMOs were added to the apical and basolateral compartments at concentrations of 1, 5 or 20 mg/mL and the cells were stimulated for 24 h. Inflammation was induced by adding 100 ng/mL TNF-α (Merck Life Science; Cat# SRP3177-50UG) and 10 ng/mL IFN-γ (InvivoGen; Cat# rcyec-hifng) to the basolateral compartment. Changes in TEER were calculated as the area under the curve (AUC) following data normalization (to the average baseline 6 h prior to stimulation) from 3 to 23 h post-stimulation (20-h duration). The AUC was based on the “Net Area” with a baseline of the minimum value of the medium control. A positive AUC indicated an increase in the TEER compared to baseline and a negative AUC indicated a decrease in TEER compared to baseline.

### 2.4 Intestinal epithelial chemokine secretion

HT29 cells were seeded with medium containing 0.1, 1 or 10 mg/mL HMOs, and this medium was replaced after 3 days. Cells exposed to 5 ng/mL LPS (Merck Life Science; Cat# L3129) were used as controls. The supernatant was harvested on day 4 for further chemokine analysis.

### 2.5 Isolation and stimulation of monocyte-derived DCs and MØs

All procedures involving the handling of human samples were carried out in accordance with the principles described in the Declaration of Helsinki and the samples were collected and analyzed following ethical approval by the Regional Ethical Committee of the Capital Region of Denmark (H-16033682). PBMCs were isolated from healthy adult donor blood by density gradient centrifugation using Lymphoprep (Stemcell Technologies; Cat#07851) and monocytes were isolated using the Easysep Human Monocyte Isolation Kit (Stemcell Technologies; Cat# 19359). To generate DCs, 1.5×10^6^ purified monocytes were cultured in flat-bottom six-well plates (Nunc; Cat# 140675) for 6 days in 3 mL RPMI-1640 medium (Merck Life Science; Cat# R5886) supplemented with 1% streptomycin, 1% penicillin, 1% l-glutamine, 10% heat-inactivated FBS, 50 ng/mL granulocyte-macrophage colony-stimulating factor (GM-CSF; PeproTech; Cat# AF-300-03) and 50 ng/mL IL-4 (PeproTech; Cat# AF-200-04). MØs were generated using the same procedure but in the absence of IL-4. After 3 days, the medium and cytokines were replenished. On day 5, differentiated DCs and MØs were resuspended in X-VIVO 15 medium (Lonza; Cat# BE02-060Q). The DCs were activated with 50 ng/mL LPS-EK (InvivoGen; Cat# tlrl-peklps), whereas MØs were activated with 50 ng/mL LPS and 50 ng/mL IFN-γ to elicit M1 polarization (37). Following the addition of individual HMOs (1, 2.5 or 5 mg/mL) the DCs and MØs were incubated for 24 h and the supernatants were collected for cytokine analysis.

### 2.6 Isolation and stimulation of CD4^+^ T cells with allogenic DCs or MØs

Naïve CD4^+^ T cells were purified from PBMCs by negative selection using the Easysep Human Naive CD4^+^ T cell Enrichment Kit (Stemcell Technologies; Cat# 19555). Briefly, antibodies targeting unwanted cell types were mixed with the PBMCs, and magnetic particles were added to bind and remove them using an EasySep Magnet (Stemcell Technologies, Cat# 180001). The remaining cell population (>95% naïve CD4^+^ T cells) was cultured with allogeneic DCs or MØs that had been activated with LPS or LPS/IFN-γ, respectively, and individual HMOs. We seeded 1×10^5^ washed MØs or DCs and 1×10^6^ naïve CD4^+^ T cells in flat-bottom, 24-well plates (Nunc; Cat# 142475) containing serum-free X-VIVO 15 medium and incubated the co-cultures for 5 days. The supernatants were then collected for cytokine analysis.

### 2.7 Cytokine and chemokine analysis

IL-1β, IL-6, IL-10, IL-12p70, IL-17A, IL-23, TNF-α, C-X-C Motif Chemokine Ligand (CXCL)8, CXCL10 and C-C Motif Chemokine Ligand (CCL)20 were measured using customized MSD 96-well electrochemiluminescence immunoassay kits (Meso Scale Discovery). IL-13 and IFN-γ were measured using enzyme-linked immunoassay (ELISA) kits (Invitrogen; Cat# BMS231-3 and EHIFNG2, respectively).

### 2.8 Statistical analysis

Graphpad Prism v9.5.0 was used to calculate the AUC for the TEER data and all statistical tests. We used an unpaired *t*-test to compare two groups and one-way or two-way analysis of variance (ANOVA) for multiple groups, followed by a *post hoc* test to determine statistical significance. Data are presented as means + standard errors of the mean (SEM) with n = 3–9 replicates depending on the assay (*p < 0.05, **p < 0.01, ***p < 0.001 and ****p < 0.0001).

## 3 Results

### 3.1 Neutral fucosylated and non-fucosylated HMOs enhance intestinal epithelial barrier integrity under non-challenged and challenged conditions

**Figure 1:**
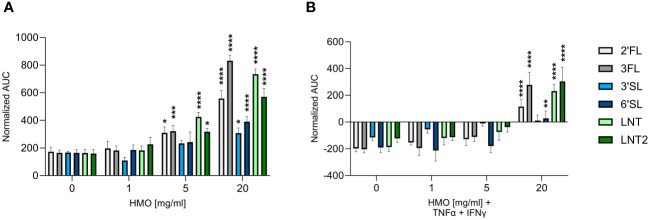
Effect of HMOs on intestinal barrier integrity. TEER was measured across Caco-2 cell monolayers exposed for 24 h to HMOs at concentrations of 1, 5 or 20 mg/mL in the absence **(A)** or presence **(B)** of TNF-α (100 ng/mL) and IFN-γ (10 ng/mL). Data are expressed as means of normalized AUC values + SEM (n = 3–9 independent experiments). Statistical significance was determined by two-way ANOVA followed by Dunnett’s multiple comparisons test (****p < 0.0001, ***p < 0.001, **p < 0.01 and *p < 0.05 compared to the unstimulated control without HMOs).

The effect of HMOs on intestinal barrier integrity was assessed by studying changes in TEER in Caco-2 cells. Apical and basolateral exposure to all six tested HMOs enhanced TEER in a dose-dependent manner from ≥ 5 mg/mL (**Figure 1A**). The fucosylated HMOs (2′FL and 3FL) as well as the neutral non-fucosylated HMOs (LNT and LNT2) had the most pronounced effect on TEER at 20 mg/mL (**Supplementary Table 1**). The two sialylated HMOs (3′SL and 6′SL) were less potent (**Figure 1A**, **Supplementary Table 1**). In the presence of an inflammatory condition induced by TNF-α and IFN-γ, the TEER declined after 24 h to ~30% below baseline, which indicates a leakier cell layer. However, the TEER was stabilized to above baseline in the presence of the pro-inflammatory cytokines plus 20 mg/mL HMOs. The effect was again most notable for the fucosylated and neutral non-fucosylated HMOs (**Figure 1B**, **Supplementary Table 1**). Together, these data indicate that structurally different HMOs have distinct effects on the host phenotype, with the neutral HMOs exerting the strongest effect on intestinal barrier integrity.

### 3.2 Sialylated HMOs enhance the epithelial chemokine response

**Figure 2:**
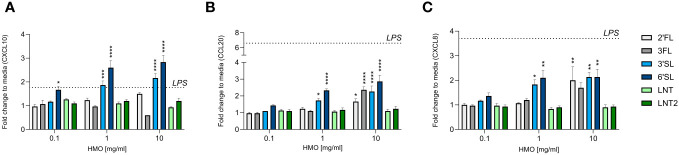
Effect of HMOs on chemokine release from intestinal epithelial cells. The charts show the induction of **(A)** CXCL10, **(B)** CCL20 and **(C)** CXCL8 release from HT29 cells triggered by different concentrations of HMOs (0.1, 1 or 10 mg/mL). Data are expressed as mean cytokine levels normalized to the unstimulated control (fold change) + SEM (n = 3 independent experiments). The mean fold-change induced by LPS compared to the control is indicated by a dashed line (see also **Supplementary Figure 1**). Statistical significance was determined by one-way ANOVA followed by Dunnett’s multiple comparisons test (****p < 0.0001, ***p < 0.001, **p < 0.01 and *p < 0.05 compared to the unstimulated control without HMOs).

The intestinal epithelium is important for the transmission of signals between the lumen (external environment) and the underlying immune cells (lamina propria) (38). Chemokines secreted by the intestinal epithelium facilitate the trafficking of immune cells to sites where they are needed (39). We measured the levels of CXCL10, CCL20 and CXCL8, which are chemoattractants for Th1 cells, Th17 cells, and neutrophils, respectively (38). HT29 cells were used as a model for the intestinal mucosal response, as these cells are known to be responsive to immunogenic stimuli including HMOs (40). The CXCL10 response was increased by the sialylated HMOs (3′SL and 6′SL) in a dose-dependent manner and reached statistically significance compared to control (**Figure 2A**). In contrast, the fucosylated (2′FL and 3FL) and neutral (LNT and LNT2) HMOs had no effect compared to the control. The CCL20 and CXCL8 responses were similar, but at the highest concentration also 2′FL and 3FL significantly increased CCL20 and CXCL8 levels (**Figures 2B, C**). LPS (5 ng/mL) was used as a positive control and increased the chemokine response, especially CCL20 and CXCL8, whereas CXCL10 levels increased only slightly (**Supplementary Figure 1**). The intestinal chemokine response was therefore found to be dependent on HMO structure, with the two sialylated HMOs exerting the strongest effect on the secretion of CXCL10, CCL20 and CXCL8.

### 3.3 An enhanced cytokine response in LPS-activated DCs is triggered by 3FL, 3′SL and 6′SL

**Figure 3:**
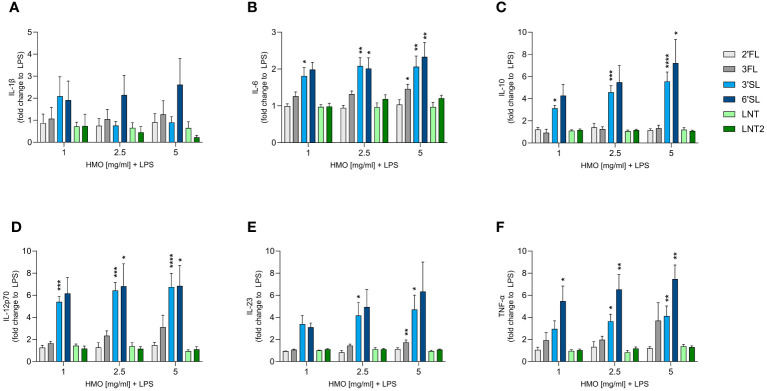
Effect of HMOs on cytokine release from LPS-activated DCs. Monocyte-derived DCs were stimulated for 24 h with LPS (50 ng/mL) in the absence or presence of individual HMOs (1, 2.5 or 5 mg/mL) before measuring the release of **(A)** IL-1β, **(B)** IL-6, **(C)** IL-10, **(D)** IL-12p70, **(E)** IL-23 and **(F)** TNF-α. Data are expressed as mean cytokine levels normalized to the LPS control group (fold-change) for each donor + SEM from 4–6 individual donors. Statistical significance was determined by one-way ANOVA followed by Dunnett’s multiple comparisons test (****p < 0.0001, ***p < 0.001, **p < 0.01 and *p < 0.05 compared to the corresponding LPS-stimulated cells).

We next assessed the direct immunomodulatory effect of the individual HMOs on innate and adaptive immune cells. Human monocyte-derived DCs were activated or not with LPS in the absence or presence of HMOs (1, 2.5 or 5 mg/mL). After 24 h, we measured the release of IL-1β, IL-6, IL-10, IL-12p70 (IL-12), IL-23 and TNF-α (**Figure 3** and **Supplementary Figure 2**). Compared to unstimulated cells, LPS significantly increased the production of all six cytokines (**Supplementary Figure 2**). The individual HMOs induced minimal cytokine secretion from non-LPS-activated DCs (**Supplementary Figure 3**). However, when DCs were conditioned with individual HMOs during activation by LPS, we observed structure-dependent enhancing effects (**Figure 3**). Specifically, 3′SL and 6′SL significantly increased the release of pro-inflammatory IL-6, IL-12p70, IL-23 and TNF-α, and anti-inflammatory IL-10 from LPS-activated DCs. Among the non-sialylated HMOs, only 3FL exerted slight modulatory effects by significantly increasing the secretion of IL-6 and IL-23, but to a much lesser extent than 3′SL and 6′SL. Overall we did not observe a significant dose-dependent response. These findings show that sialylated HMOs added to LPS-activated DCs augmented the production of both pro-inflammatory and anti-inflammatory cytokines that drive Th1 and Th17 responses as well as regulatory immune responses.

### 3.4 Sialylated HMOs and 3FL increase cytokine responses in LPS-activated M1 MØs

**Figure 4:**
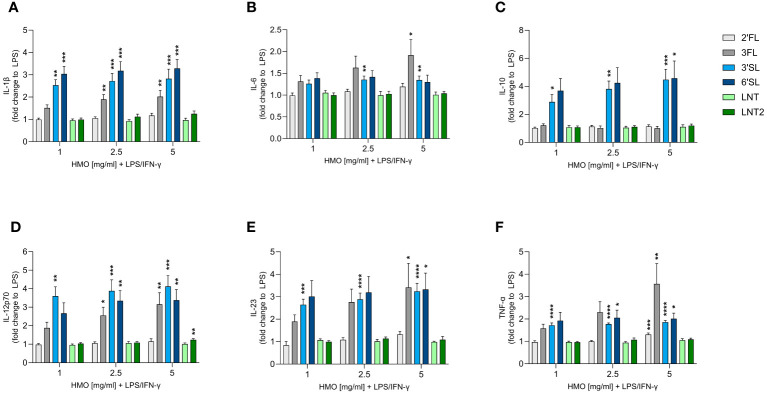
Effect of HMOs on cytokine release from LPS-activated M1 MØs. Monocyte-derived MØs were stimulated for 24 h with LPS (50 ng/mL) and IFN-γ (50 ng/mL) in the absence or presence of individual HMOs (1, 2.5 or 5 mg/mL) before measuring the release of **(A)** IL-1β, **(B)** IL-6, **(C)** IL-10, **(D)** IL-12p70, **(E)** IL-23 and **(F)** TNF-α. Data are expressed as mean cytokine levels normalized to the LPS control group (fold-change) for each donor + SEM from 4–6 individual donors. Statistical significance was determined by one-way ANOVA followed by Dunnett’s multiple comparisons test (****p < 0.0001, ***p < 0.001, **p < 0.01 and *p < 0.05 compared to the corresponding LPS-stimulated cells).

Although monoculture experiments with HMOs and murine macrophages have been reported, the effect of HMOs on human monocyte-derived MØs is unknown (41). We therefore assessed the immunomodulatory effect of single HMOs on IFN-γ-primed classically activated M1 MØs to assess their impact on immune responses during an inflammatory state (**Figure 4** and **Supplementary Figure 4**). The activation of M1 MØs by LPS significantly increased the secretion of IL-1β, IL-6, IL-10, IL-12p70, IL-23 and TNF-α compared to non-activated MØs. IL-1β, IL-6, IL-12p70, IL-23 and TNF-α were secreted at much higher levels by LPS-activated M1 MØs than LPS-activated DCs, although the activation pattern was the same (**Supplementary Figure 4**). Overall, the sialylated HMOs increased the secretion of all tested cytokines and to a greater extent than the other HMOs except 3FL (**Figure 4**). Specifically, 3FL, 3′SL and 6′SL significantly increased the secretion of IL-1β, IL-12p70, IL-23 and TNF-α, whereas only the sialylated HMOs significantly increased the secretion of IL-10. Moreover, IL-6 secretion was significantly increased solely by 3FL and 3′SL The other three HMOs showed negligible modulatory effects, with the highest doses of 2′FL and LNT2 only slightly, albeit significantly, increasing the levels of TNF-α and IL-12p70, respectively. Generally, there was no significant dose-dependent effect. These results demonstrate that sialylated HMOs (and to some extent 3FL) added to LPS-activated classical M1 MØs enhance the production of pro-inflammatory and anti-inflammatory cytokines as observed for HMO-conditioned LPS-activated DCs.

### 3.5 LPS-activated DCs and M1 MØs differentially modulate allogenic T helper responses when conditioned with 3FL, 3′SL or 6′SL

**Figure 5:**
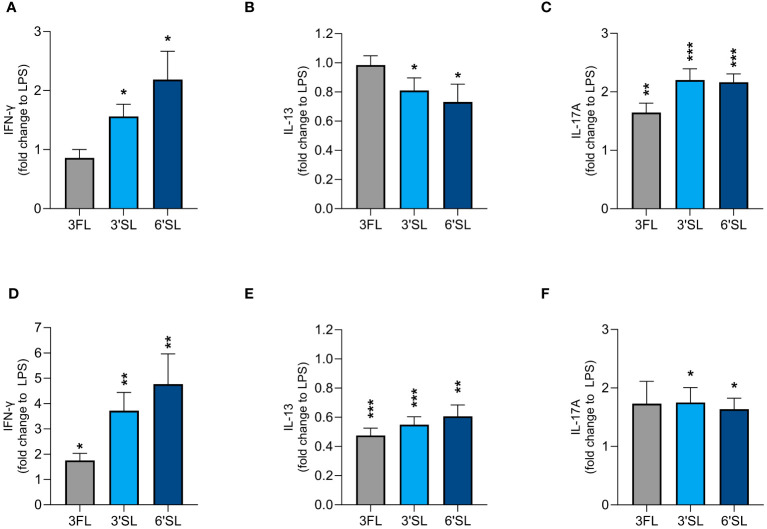
Effect of HMO-conditioned LPS-activated DCs **(A–C)** or M1 MØs **(D–F)** on cytokine release from CD4^+^ T cells. Monocyte-derived DCs and MØs were activated with LPS and LPS/IFN-γ, respectively, in the presence or absence of individual HMOs (1 mg/mL) for 24 h, followed by co-culture with allogenic naïve CD4^+^ T cells for a further 5 days. Data are expressed as mean cytokine levels normalized to the corresponding LPS control group (fold-change) for each donor + SEM from n = 9 donor combinations. Statistical significance was determined by applying an unpaired t-test (***p < 0.001, **p < 0.01 and *p < 0.05 compared to corresponding LPS-stimulated control cells).

We found that 3′SL, 6′SL and 3FL exerted immunomodulatory effects on DCs and M1 MØs. To explore whether HMO-conditioned DCs and MØs modulate CD4^+^ T cell responses, we activated DCs and M1 MØs in the presence of 3′SL, 6′SL or 3FL for 24 h before co-culture with allogeneic naïve CD4^+^ T cells. After co-culture for 5 days, we measured the production CD4^+^ T cell effector cytokines including IFN-γ (Th1), IL-13 (Th2), and IL-17A (Th17) (**Figure 5** and **Supplementary Figure 5**). CD4^+^ T cells co-cultured with non-activated DCs produced IFN-γ, IL-13 and some IL-17A (**Supplementary Figures 5A–C**). In comparison, CD4^+^ T cells co-cultured with non-activated MØs produced minimal IL-13 and IL-17A and no IFN-γ (**Supplementary Figures 5D–F**). LPS-activated DCs and MØs significantly increased the production of IFN-γ, IL-13 and IL-17A from T cells compared to non-activated DCs or MØs (**Supplementary Figure 5**).

LPS-activated DCs conditioned with 3′SL and 6′SL significantly increased the secretion of IFN-γ and IL-17A by CD4^+^ T cells and significantly reduced the secretion of IL-13. In contrast, LPS-activated DCs conditioned with 3FL significantly increased the secretion of IL-17A but not IFN-γ and did not influence the secretion of IL-13 (**Figures 5A–C**). HMO-conditioned LPS-activated M1 MØs had a slightly greater impact on Th1 and Th2 differentiation, with all three HMOs significantly increasing IFN-γ secretion and significantly reducing the secretion of IL-13 compared to LPS alone (**Figures 5D, E**). All three HMOs also increased the secretion of IL-17A from CD4^+^ T cells co-cultured with M1 MØs, with a statistically significant difference observed for 3′SL and 6′SL (**Figure 5F**). Compared to 3FL, the two sialylated HMOs had a greater impact on the enhanced Th1 response. Collectively, these findings show that DCs and M1 MØs conditioned with 3′SL or 6′SL enhanced Th1 and Th17 responses, and lowered Th2 responses, whereas 3FL had less pronounced effects.

## 4 Discussion

HMOs directly support the maturation of the intestinal barrier and immune system in early life (7, 8). In this study, we focused on HMOs representing different structural classes to gain more insight into their structure-dependent functions on the intestinal epithelium as well as innate and adaptive immunity. We found that individual HMOs affected the intestinal epithelial cell barrier function and chemokine secretion, directly influenced cytokine secretion from innate immune cells (DCs and MØs), and subsequently modulated T cell effector functions, in a structure-dependent manner.

We first studied the effect of the individual HMOs on intestinal barrier integrity and found that fucosylated HMOs (3FL especially, and 2′FL) and neutral non-fucosylated HMOs (LNT and LNT2) displayed the strongest effect by enhancing the barrier both in the presence and absence of a challenge. Notably, we only saw an effect when the intestinal epithelium was exposed to the HMOs on both sides, in contrast to luminal or apical exposure alone. In early life, the intestine is more permeable, which may allow a higher proportion of HMOs to enter the circulation, thereby providing access to the basolateral compartment (18). The effect was most pronounced at the highest dose (20 mg/ml), which exceeds the levels of HMOs found in human milk [5-15g/L (42)], and expectedly also in the circulation of infants. However, the applied doses were selected with the aim of seeing potential effects and compare the individual HMOs. A similar dose has also been applied in previous studies testing HMOs in Caco-2 cells (5, 20). In one of these, it was also found that 2′FL in a blend of six HMOs (2′FL, 3′SL, 6′SL, LNT, difucosyllactose and lacto-*N*-neotetraose) is the key driver of enhanced intestinal barrier integrity and protection following exposure to pro-inflammatory cytokines (5). LNT also had an effect, but it was less potent than 2′FL (5). In our study, we also tested 3FL, which had a pronounced effect compared to the other HMOs. Thus, in line with earlier studies, our findings confirm that fucosylated and neutral non-fucosylated HMOs also support intestinal barrier integrity during an inflammatory insult.

We investigated the impact of HMOs on DC and M1 MØ monocultures, as well as co-cultures of both cell types with allogeneic CD4^+^ T cells. Given that these immune cells were derived from PBMCs isolated from healthy donors, these models offer insight into the potential systemic effects of HMOs passing into the circulation. We found that the two sialylated HMOs (3′SL and 6′SL) had the most pronounced effect, stimulating the secretion of IL-10, IL-12p70 and IL-23 from LPS-activated DCs and M1 MØs, whereas the secretion of IL-1β was only significantly enhanced in LPS-activated M1 MØs. To the best of our knowledge, these are novel findings in the context of individual HMOs. Notably, we did not observe a significant dose-dependent effect, which could be due to saturation of DC and MØ receptor-mediated signaling responses already at the lowest dose of HMOs we tested (1 mg/mL) (43). We therefore used the same concentration (1 mg/mL) in our co-culture models with naïve CD4^+^ T cells.

Consistent with our monoculture results, we found that 3′SL and 6′SL enhanced the secretion of IFN-γ and IL-17A and reduced the secretion of IL-13 by naïve CD4^+^ T-cells co-cultured with LPS-activated DCs or M1 MØs, demonstrating that both sialylated HMOs induced Th1 and Th17 differentiation while inhibiting Th2 differentiation. Notably, 6′SL appeared to be a slightly more potent inducer of Th1 responses than 3′SL. In comparison, 3FL primed the same pattern of Th differentiation but to a lesser degree, and primarily in co-cultures of CD4^+^ T cells and M1 MØs. These findings are interesting because the neonatal immune system is considered tolerogenic, with enhanced immunosuppressive and tissue-protective mechanisms to maintain balance and prevent collateral damage (44). However, neonatal DCs, MØs and monocytes show impaired antigen-presenting functions, cytokine secretion and T cell stimulation. Consequently, neonatal T cells skew toward Th2 rather than Th1 responses, and coupled with their inexperienced adaptive immune functions, this renders neonates more vulnerable to viral and bacterial infections (45–47). Our findings indicate that certain HMOs, in this case primarily 3′SL and 6′SL, may directly interact with and modulate DC and MØ functions to subsequently support Th1 responses.

The Th17 cell lineage is often described as the third major subset of effector T cells, and Th17 responses during homeostatic and regulated conditions play a critical role in antibacterial and antifungal immunity at epithelial surfaces (48, 49). Importantly, preterm infants are more susceptible to infections at mucosal surfaces, probably in part due to the developmental limitations of neonatal Th17 functions (50). To our knowledge, our study is the first to demonstrate that sialylated HMOs promote Th17-skewed responses in CD4^+^ T cells co-cultured with DCs or M1 MØs. Similar effects of 3′SL and/or 6′SL in clinical settings may reduce the risk of mucosal infections in neonates.

Our findings that sialylated HMOs can modulate the activity of DCs and M1 MØs in monoculture and in co-culture with CD4^+^ T cells by enhancing Th1 and Th17 responses align with previous reports. The acidic HMO fraction (most likely to be rich in sialylated structures) of pooled HMOs isolated from breastmilk increased IFN-γ production from cord blood-derived mononuclear cells (34) and 3′SL was previously shown to induce mesenteric lymph node-derived DCs to produce cytokines driving Th1 and Th17-dependent inflammation in an *il10^–/–^* mouse model of spontaneous colitis (36). Moreover, 3FL, 3′SL and 6′SL enhanced IFN-γ secretion from PBMCs activated with anti-CD3/CD28 antibodies although statistical significance was only achieved for 3FL (35). Notably, 2′FL and 6′SL failed to affect the LPS-induced maturation of DCs and DC cytokine secretion when the HMOs were applied during 6 days of monocyte-to-DC differentiation, suggesting that HMOs have less of an impact on this immunomodulatory process (51). However, pooled human HMOs (isolated from breastmilk) reduced rather than increased the LPS-induced production of IL-12p70, IL-6 and TNF-α from human monocyte-derived DCs, which subsequently reduced Th1 differentiation and IFN-γ secretion (33). This contrasting finding may reflect the small proportion (10–14%) of the HMO pool in human breastmilk made up of sialylated HMOs (52), thus possibly diluting their impact. Moreover, other HMO structures beyond the six included in the present study may have distinct immunomodulatory properties, and some may reduce rather than enhance cytokine secretion from LPS-activated DCs. The aim of the present study was to focus on the host phenotypes induced by individual HMOs, and future/subsequent studies should focus on fully characterizing the underlying mechanisms by identifying the receptors on intestinal epithelial cells and the monocyte-derived DCs and MØ that HMOs engage to promote or suppress signaling. It is already known that different structural classes of HMOs can bind with varying affinity to classes of lectin (glycan-binding protein) receptors expressed on monocytes and other immune cell types. For instance, DC-specific intercellular adhesion molecule-3-grabbing non-integrin (DC-SIGN) and sialic acid-binding, immunoglobulin-like lectins (siglecs) have been suggested as receptors for fucosylated and sialylated HMOs, respectively (53). Toll-like receptor (TLR)-4, expressed on the surface of both monocytes and intestinal epithelial cells, has been implicated as a mediator of HMO-induced immune responses (33, 36). Thus, it is possible that 3′SL and 6′SL modulate the cytokine release from DCs and M1 MØs by interacting with specific siglecs. To add more insight into the underlying cellular mechanisms by which the individual HMOs interact with the host, applying transcriptomic and proteomic approaches may be supportive (54).

LPS contaminated HMOs have been addressed as a major concern, suggesting any host effects are caused by LPS rather than the HMOs. In one study the apparent TLR-4 mediated immunomodulatory effects of 3’SL on human monocyte-derived DCs were attributed to LPS contamination (10 EU/mg), thus warranting caution and a need for assessing LPS contamination levels when studying e.g., TLR-mediated effects of HMOs (55). The HMOs studied in the present study had a very low endotoxin level (<0.009 EU/mg, **Table 1**), which is not in the range expected to significantly confound immunological responses, as shown previously (35, 55). Importantly, HMO supplementation to non-activated DCs stimulated minimal cytokine secretion compared to LPS-activated DCs (**Supplementary Figure 3**).

We also assessed the impact of HMOs on chemokine secretion from intestinal epithelial cells. The intestinal epithelium engages in crosstalk with the innate and adaptive immune systems by secreting chemokines, and thus plays a key role in the regulation of mucosal immune responses (14). We measured the secretion of CXCL10, CCL20 and CXCL8, and found that mainly the sialylated HMOs induced the secretion of all three chemokines, with 6′SL having a slightly greater impact than 3′SL. CXCL10 is a chemoattractant for Th1 cells that acts through CXCR3 expressed on (among others) CD4^+^ Th1 cells and is induced by IFN-γ (56). CCL20 is a chemoattractant for CCR6^+^ cells such as DCs, B cells and CD4^+^ T cells (especially Th17 cells) and CD8^+^ T cells (57, 58). CCL20 also has antimicrobial activity, which reflects its ability to form a cationic antimicrobial peptide that can penetrate bacterial membranes and bind to intracellular components (59). Notably, the upregulation of *CCL20* and *CXCL8* gene expression has been demonstrated by the transcriptomic analysis of HT29 cells exposed to 3′SL or pooled HMOs, but the structure-dependent effects of individual HMOs were not reported (40).

Given the extensive crosstalk between intestinal epithelial cells and immune cells, it would be informative to investigate the impact of individual HMOs in co-cultures of these cell types. Such models more closely mimic mucosal immune responses, whereas here we have focused on systemic immunomodulatory effects. In a recent study using co-cultures of HT29 human colon epithelial cells conditioned with CpG (a synthetic TLR9 ligand) and PBMCs activated with anti-CD3/CD28 antibodies, 2′FL and 3FL were shown to promote stronger immunomodulatory effects linked to Th1 and regulatory T (T_reg_) cell responses than 3′SL and 6′SL (35). Similarly, DCs exposed to 2′FL and CpG-conditioned HT29 cells gained an enhanced ability to promote the secretion of IFN-γ and IL-10 by CD4^+^ T cells (60). Moreover, 2′FL tended to reduce Th2 responses when naïve CD4^+^ T cells were incubated with DCs that had been co-cultured with HT29 cells in the presence of polyinosinic-polycytidylic acid, a synthetic dsRNA analog (61). Future studies evaluating HMOs representing different structural classes should consider model systems closer to the infant state, such as primary intestinal epithelial cells derived from infants or mucosal-derived immune cells. This would provide more insight into the potential structure-dependent effect HMOs on mucosal immunity during early life. Finally, given the complex mixture of HMOs present in breastmilk, it is important to test HMO mixes from different structural groups in these model systems to identify any synergistic and/or antagonistic effects.

**Figure 6:**
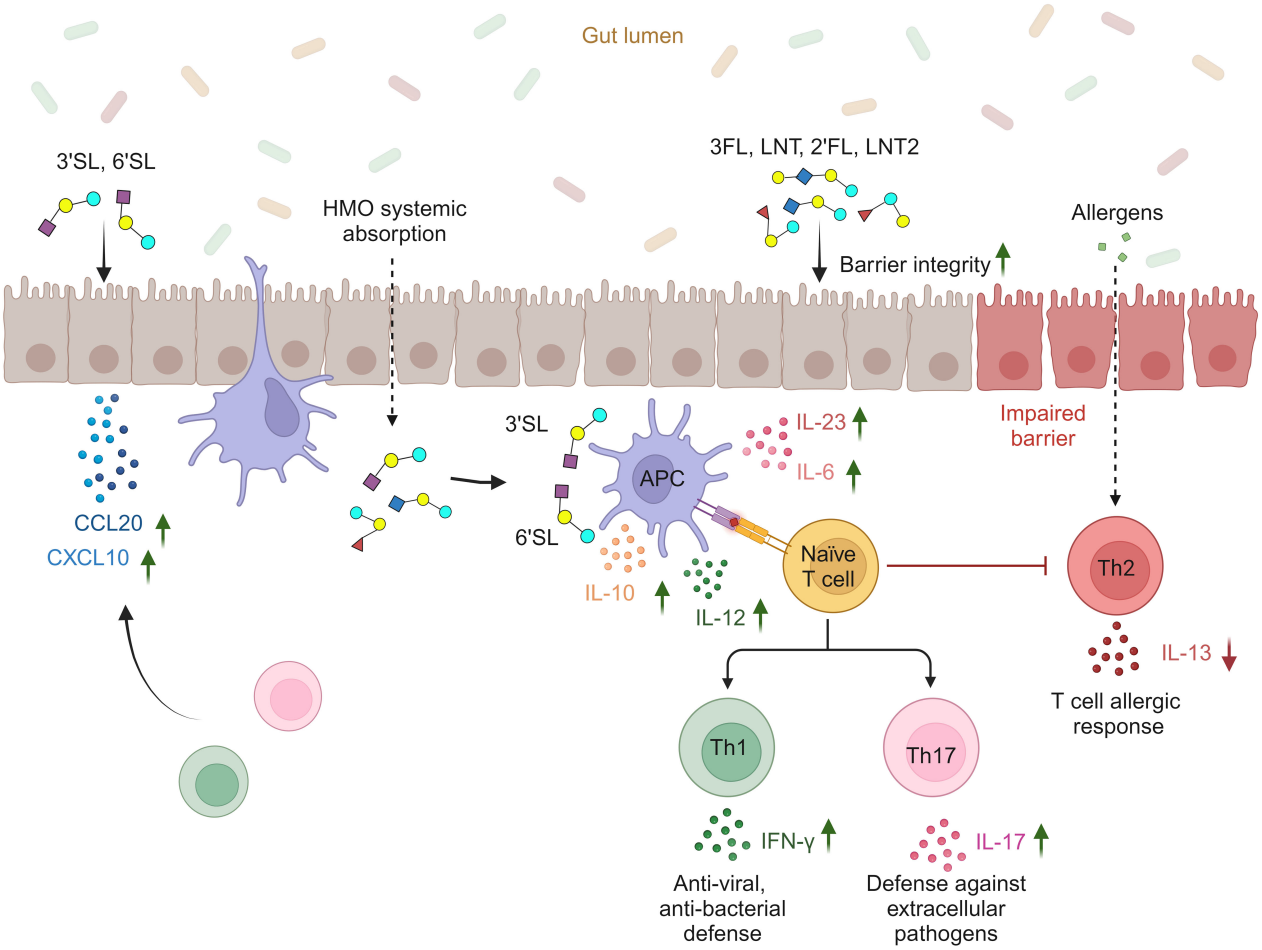
Proposed structure-dependent effects of individual HMOs on intestinal barrier integrity, intestinal epithelial secretion of Th1- and Th17-homing chemokines, cytokine release from APCs (DCs and M1 MØs), and subsequent priming of Th1 and Th17 responses (created with BioRender.com).

In conclusion, our findings highlight the potential structure-dependent effects of six individual HMOs on the intestinal barrier and local and/or systemic immune cells (**Figure 6**). The fucosylated (2′FL and 3FL) and neutral non-fucosylated (LNT and LNT2) HMOs primarily support intestinal barrier integrity, whereas the sialylated (3′SL and 6′SL) HMOs enhance mucosal and systemic immune responsiveness by promoting DC and MØ-mediated Th1 and Th17 responses and reducing Th2 responses. Furthermore, 3′SL and 6′SL affect chemokine networks in the intestinal epithelium and may facilitate the recruitment of Th1 and Th17 subtypes to the intestinal submucosa. Concurrently, the enhanced secretion of IL-10 by DCs and MØs helps to ensure the immune responses are balanced and not over-reactive. Combining these findings with the fact that HMOs are a significant constituent of breastmilk with more than 150 different structures, our results suggest that individual HMOs play distinct and important roles that support development in early life.

## Data availability statement

The original contributions presented in the study are included in the article/**Supplementary Material**. Further inquiries can be directed to the corresponding author.

## Author contributions

EB: Conceptualization, Formal analysis, Investigation, Methodology, Writing – original draft, Writing – review and editing. DL: Conceptualization, Formal analysis, Investigation, Methodology, Writing – original draft, Writing – review and editing. MT: Investigation, Methodology, Writing – review and editing. SH: Investigation, Methodology, Writing – review and editing. KP: Writing – review and editing. SJ: Conceptualization, Formal analysis, Investigation, Methodology, Writing – original draft, Writing – review and editing.
