# Supplementary material for: Human milk oligosaccharides differentially support gut barrier integrity and enhance Th1 and Th17 cell effector responses in vitro
Source: Front Immunol. 2024 Mar 6;15:1359499. doi: 10.3389/fimmu.2024.1359499 (PMC10950922; doi:10.3389/fimmu.2024.1359499)
Supplement: Supplementary file 1 [file DataSheet_1.docx]

Supplementary Material

# Supplementary Tables

| **HMOs**  **(20 mg/mL)** | **ΔMean** | **Statistic** | **ΔMean** | **Statistic** |
| --- | --- | --- | --- | --- |
|  | **(÷Challenge)** | **(÷Challenge)** | **(+Challenge)** | **(+Challenge)** |
| 3FL vs. 3′SL | 525 | **** | 266 | 0.0542 |
| 3FL vs. 6′SL | 441 | **** | 250 | ns |
| 3FL vs 2′FL | 273 | *** | 162 | ns |
| 3FL vs. LNT2 | 262 | ** | -27 | ns |
| 3FL vs. LNT | 98 | ns | 46 | ns |
| LNT vs. 3′SL | 427 | **** | 220 | ns |
| LNT vs. 6′SL | 344 | **** | 204 | ns |
| LNT vs. 2′FL | 176 | ns | 116 | ns |
| LNT vs. LNT2 | 165 | ns | -73 | ns |
| LNT2 vs. 3′SL | 263 | * | 293 | 0.053 |
| LNT2 vs. 6′SL | 179 | ns | 276 | ns |
| LNT2 vs. 2′FL | 11 | ns | 188 | ns |
| 2′FL vs. 3′SL | 252 | ** | 105 | ns |
| 2′FL vs. 6′SL | 168 | ns | 88 | ns |
| 6′SL vs. 3′SL | 84 | ns | 17 | ns |

**Supplementary Table 1**. Statistical comparison of TEER AUC values representing the effects of single HMOs at concentrations of 20 mg/mL. Mean differences (∆Mean) are listed and ranked based on HMOs with the largest ΔMean without challenge (–Challenge). Statistical significance was determined by one-way ANOVA followed by Tukey’s multiple comparisons test (*****p* < 0.0001, ****p* < 0.001, ***p* < 0.01 and **p* < 0.05).

# Supplementary Figures

**
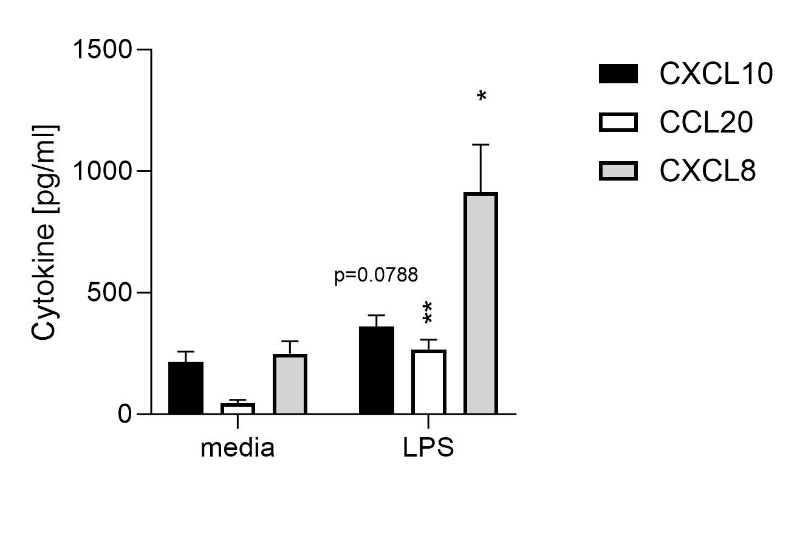
**

**Supplementary Figure 1**. Effect of 5 ng/mL LPS on chemokine release by HT29 cells. Data are expressed as means + SEM (n = 3 independent experiments). Statistical significance was determined by applying an unpaired t-test (***p* < 0.01 and **p* < 0.05 compared to medium control).


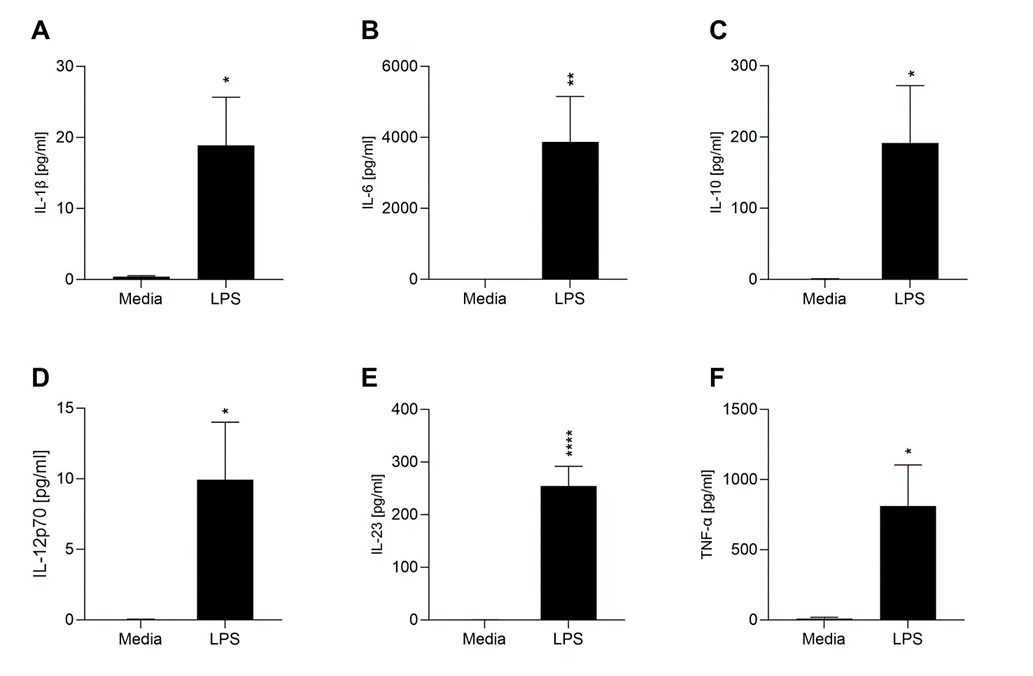


**Supplementary Figure 2**. Effect of LPS on cytokine production by DCs. Monocyte-derived DCs were stimulated for 24 h with LPS (50 ng/mL) or left untreated before measuring the release of (**A**) IL-1β, (**B**) IL-6, (**C**) IL-10, (**D**) IL-12p70, (**E**) IL-23 and (**F**) TNF-α. Data are expressed as mean cytokine levels + SEM for at least six individual donors. Statistical significance was determined by applying an unpaired t-test (*****p* < 0.0001, ***p* < 0.01, **p* < 0.05 compared to medium control).


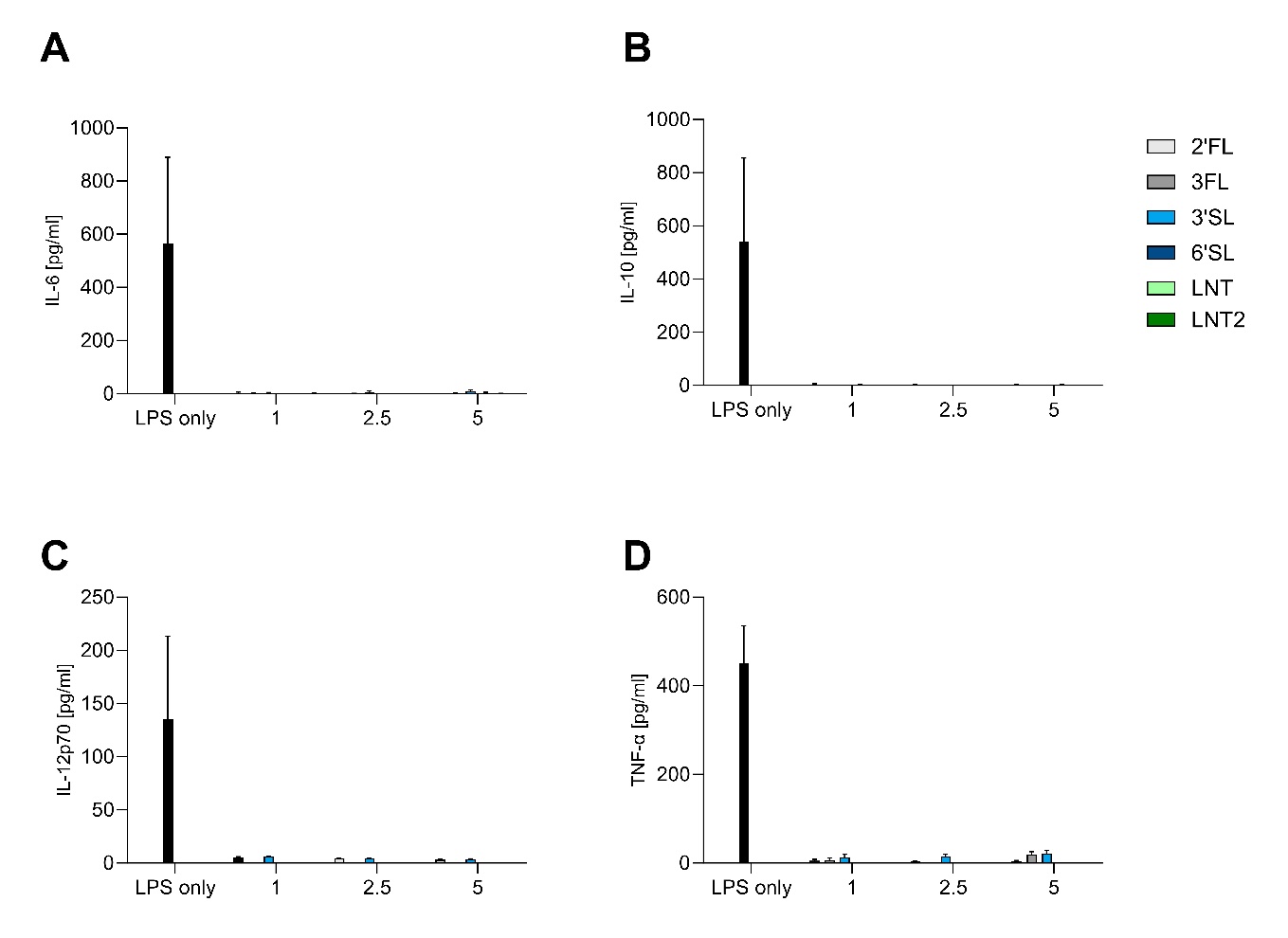


**Supplementary Figure 3**. Effect of HMOs on cytokine release from non-LPS-activated DCs. Monocyte-derived DCs were stimulated for 24 h with individual HMOs (1, 2.5 or 5mg/ml) or LPS (50 ng/mL) before measuring the release of (**A**) IL-6, (**B**) IL-10, (**C**) IL-12p70 or (**D**) TNF-α. Data are expressed as mean cytokine levels + SEM for three individual donors.

**
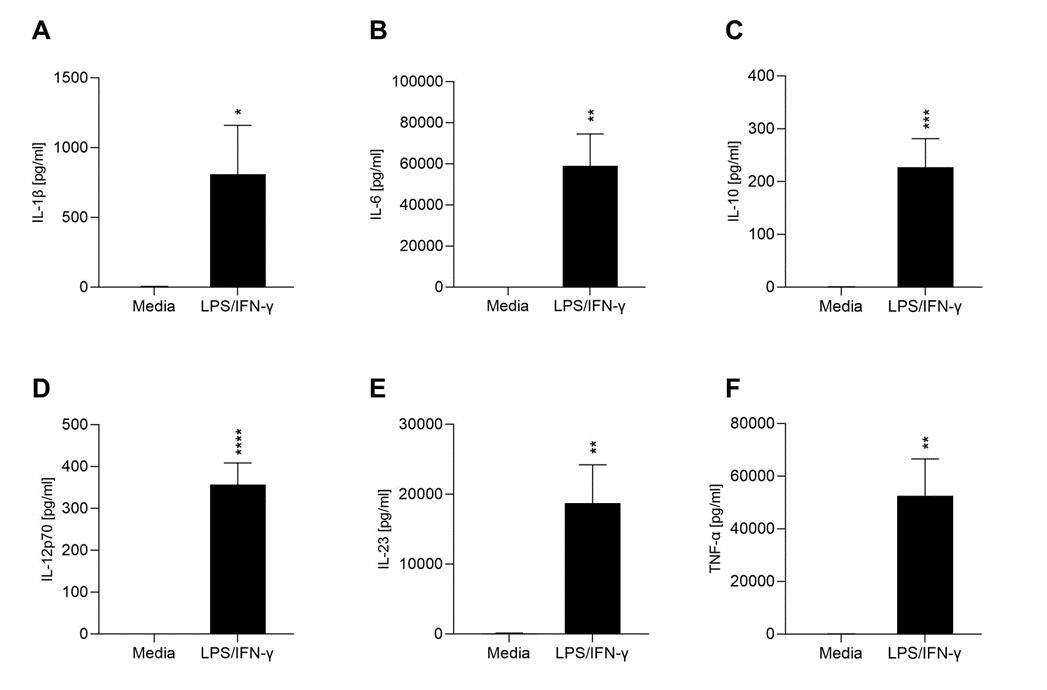
**

**Supplementary Figure 4**. Effects of LPS on cytokine production by M1 MØs. Monocyte-derived MØs were cultured for 24 h in the absence or presence of LPS and IFN-γ (50 ng/mL each) before measuring the release of (**A**) IL-1β, (**B**) IL-6, (**C**) IL-10, (**D**) IL-12p70, (**E**) IL-23 and (**F**) TNF-α. Data are expressed as mean cytokine levels + SEM for at least six individual donors. Statistical significance was determined by applying an unpaired t-test (*****p* < 0.0001, ***p* < 0.01, **p* < 0.05 compared to medium control).


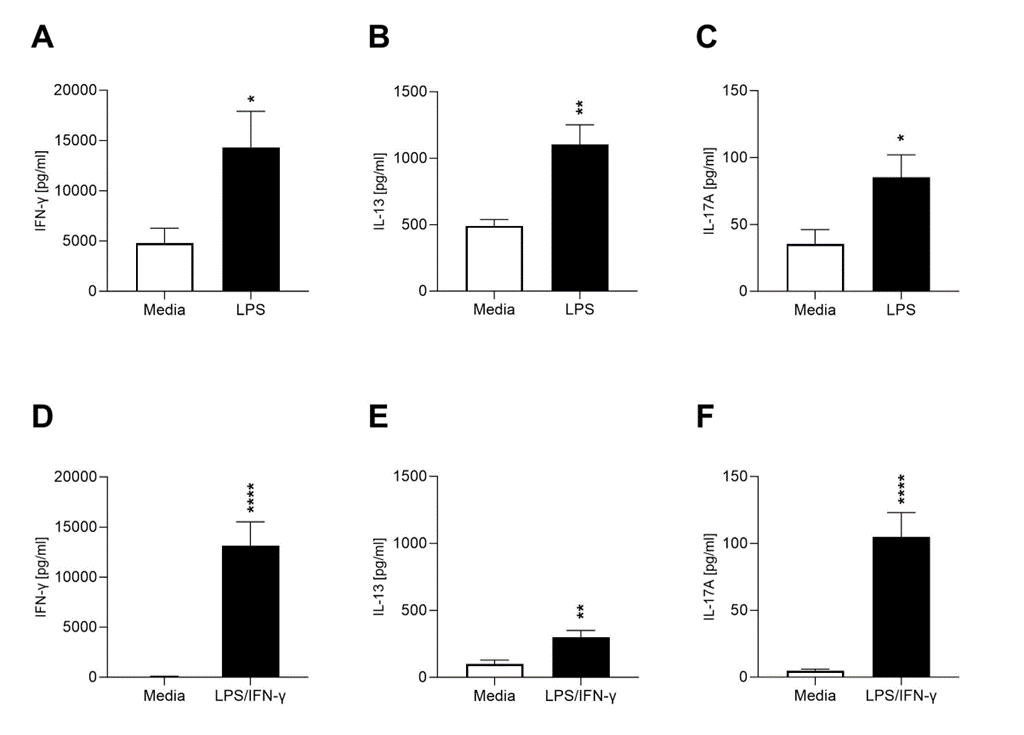


**Supplementary Figure 5**. Effect of HMO-conditioned LPS-activated DCs (**A-C**) or M1 MØs (**D-F**) on cytokine release from CD4^+^ T cells. Monocyte-derived DCs were activated with LPS (50 ng/mL) and monocyte-derived M1 MØs were activated with LPS and IFN-γ (50 ng/mL each) for 24 h, followed by co‑culture with allogenic naïve CD4^+^ T cells for a further 5 days before measuring the levels of (**A,D**) IFN-γ, (**B,E**) IL-13 and (**C,F**) IL-17A. Data are expressed as mean cytokine levels + SEM from n = 9 individual donor combinations. Statistical significance was determined by applying an unpaired t-test (*****p* < 0.0001, ***p* < 0.01, **p* < 0.05 compared to medium control).
